# ctDNA detected by ddPCR reveals changes in tumour load in metastatic malignant melanoma treated with bevacizumab

**DOI:** 10.1038/s41598-019-53917-5

**Published:** 2019-11-25

**Authors:** Rakel Brendsdal Forthun, Randi Hovland, Cornelia Schuster, Hanne Puntervoll, Hans Petter Brodal, Heidi Maria Namløs, Lars Birger Aasheim, Leonardo A. Meza-Zepeda, Bjørn Tore Gjertsen, Stian Knappskog, Oddbjørn Straume

**Affiliations:** 1 Department of Internal Medicine, Hematology Section, Haukeland University Hospital, Bergen, Norway; 2 Department of Medical Genetics, Haukeland University Hospital, Bergen, Norway; 3 Department of Biosciences, University of Bergen, Bergen, Norway; 4 Centre of Cancer Biomarkers, CCBIO, Department of Clinical Science, University of Bergen, Bergen, Norway; 5 Department of Oncology, Haukeland University Hospital, Bergen, Norway; 6 Department of Tumour Biology, Institute for Cancer Research, Oslo University Hospital, Oslo, Norway; 7 Norwegian Cancer Genomics Consortium, Institute for Cancer Research, The Norwegian Radium Hospital/Oslo University Hospital, Oslo, Norway; 8 Genomics Core Facility, Department of Core Facilities, Oslo University Hospital, Oslo, Norway; 9 K.G. Jebsen Center for Genome Directed Cancer Therapy, Department of Clinical Science, University of Bergen, Bergen, Norway

**Keywords:** Prognostic markers, Tumour biomarkers

## Abstract

Bevacizumab is included in an increasing number of clinical trials. To find biomarkers to predict and monitor treatment response, cancer and angiogenesis relevant mutations in tumour and circulating tumour DNA (ctDNA) were investigated in 26 metastatic melanoma patients treated with bevacizumab. Patients with >1% *BRAF/NRAS* ctDNA at treatment start had significantly decreased progression free survival (PFS) and overall survival (OS) (PFS: p = 0.019, median 54 vs 774 days, OS: p = 0.026, median 209 vs 1064 days). Patients with >1% *BRAF/NRAS* ctDNA during treatment showed similar results (PFS: p = 0.002, OS: p = 0.003). ≤1% *BRAF/NRAS* ctDNA and normal lactate dehydrogenase (LDH) levels both significantly predicted increased response to treatment, but *BRAF/NRAS* ctDNA was better at predicting response compared to LDH at treatment start (OR 16.94, p = 0.032 vs OR 4.57, p = 0.190), and at predicting PFS (HR 6.76, p = 0.002) and OS (HR 6.78, p = 0.002) during therapy. ctDNA *BRAF* p.V600D/E/K and *NRAS* p.G12V/p.Q61K/L/R were better biomarkers for response prediction than *TERT* promoter mutations (OR 1.50, p = 0.657). Next generation sequencing showed that all patients with ≥2 mutations in angiogenesis-relevant genes had progressive disease, but did not reveal other biomarkers identifying responders. To conclude, ctDNA and LDH are useful biomarkers for both monitoring and predicting response to bevacizumab.

## Introduction

Tumour burden in malignant melanoma patients is in general followed using computer tomography (CT) to detect relapse or progression of disease. This method gives reliant results, but is costly and time consuming and can become a bottleneck in the hospital system. Liquid biopsies have therefore been proposed to be applicable in malignant melanoma to detect both response to therapy and early relapse^1^. These non-invasive biopsies use body fluids such as blood, urine and saliva to find circulating tumour cells (CTCs), circulating tumour DNA (ctDNA), tumour associated endothelial cells or tumour derived microvesicles. Sequencing of the tumour DNA permits detection of mutations classifying malignant cells in each patient. Tracking of these mutations in plasma can be performed using either PCR based methods or next generation sequencing (NGS) specifically targeting one mutation, or by defined gene panels that can be used on a larger patient subgroup.

For cutaneous malignant melanoma, mutations in *BRAF* and *NRAS* are found in approximately 50% and 20% of all patients^2^, respectively, and are regarded as early events in tumourigenesis. These mutations therefore represent attractive targets for monitoring tumour burden by ctDNA in blood samples. Another target is mutations in the *TERT* promoter. These are found in 60–70% of malignant melanomas^3,4^ and are correlated with adverse outcome^5^, particularly when combined with *BRAF* or *NRAS* mutations^6,7^.

In this study 26 malignant melanoma patients with metastatic, non-resectable tumours were treated with bevacizumab, a monoclonal antibody specifically targeting VEGF-A^8^. The drug is currently investigated in several clinical trials, including melanoma, colorectal, ovarian and non-small cell lung cancer (ClinicalTrials.gov Identifiers NCT00790010, NCT03743428, NCT02884648, NCT03836066, respectively). In melanoma the drug gives significantly increased disease-free interval as monotherapy in an adjuvant setting^9^. Studies show that high serum concentration of Activin A^10^ is associated with objective response to bevacizumab. Thus far, ctDNA has been detected in patients treated with bevacizumab^11^, but quantitative measurements have not been done. To identify biomarkers that are easy to measure we therefore performed mutational analysis using NGS on tumour biopsies and plasma samples, and digital droplet PCR (ddPCR) on plasma samples. We aimed to determine whether patients’ mutational profile, ctDNA levels or lactate dehydrogenase (LDH) levels could serve as predictive markers for response to bevacizumab, prognostic markers for progression free survival (PFS) and overall survival (OS) or if changes in ctDNA or LDH during treatment could serve as pharmacodynamic markers. As care must be taken when using single gene analysis as measure for tumour burden, the investigated mutation should be a primary hit present in all tumour cells. We therefore compared the ctDNA fraction of *TERT* promoter mutations to *BRAF*/*NRAS*.

## Results

### Survival and response

**Figure 1:**
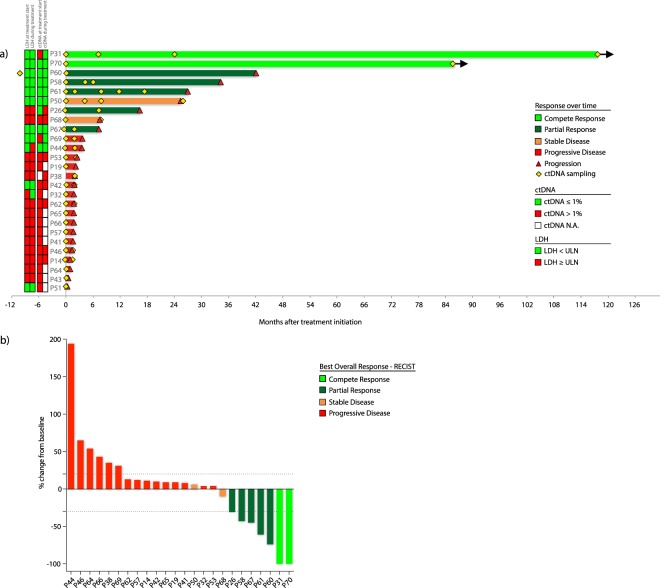
Best overall response and progression free survival. (**a)** Progression free survival is indicated for all 26 patients. Plasma samples were collected as indicated (yellow diamonds). Two patients were complete responders (light green) and are still in remission seven (P70) and ten years (P31) after treatment start. Partial responders (dark green, n = 5), patients with stable disease (orange, n = 2) and progressive disease (red, n = 17) received 1–74 cycles of treatment before progression (red triangles). LDH and *BRAF/NRAS* ctDNA fractional abundances before treatment and during treatment (first sample point after treatment initiation) are indicated to the left of the Y-axis. (**b**) Best overall response to bevacizumab for 25 patients who had undergone at least one tumour assessment measured as the change from baseline in the sum of the largest diameters of each target lesion. P43 progressed clinically before first tumour assessment and is not shown. According to RECIST, progressive disease was defined by occurrence of new lesions in some patients in spite of stable target lesions. Stapled lines indicate cut-off for RECIST scores. PFS and BOR by August 2011 were previously published^12^, this figure depicts data updated per June 2017.

Twenty-six patients with inoperable metastatic melanoma were treated with bevacizumab as monotherapy (Table 1, *Methods section*). Nine patients responded to treatment (34.6%); complete response was achieved in two patients (CR; 7.7%), partial response in five patients (PR; 19.2%) and stable disease longer than 6 months in two patients (SD; 7.7%). By June 2017, both complete responders were alive and disease free more than seven (P70) and 10 years (P31) after treatment was stopped (Fig. 1a). Seventeen patients progressed within 6 months of treatment (best overall response is shown in Fig. 1b). Median PFS was 64 days and median OS was 265 days. Details on the clinical trial are previously published^12^.

**Table 1:** Patient characteristics.

Characteristics	Study cohort (n = 26)
Age, years	
Median	63
Range	29–77
Sex – No. (%)	
Male	15 (58%)
Female	11 (42%)
Stage – No. (%)	
M1a	1 (4%)
M1b	4 (15%)
M1c	21 (81%)
LDH > ULN – No. (%)	
No	12 (46%)
Yes	14 (54%)
WHO performance status – No. (%)	
0	22 (85%)
1	4 (15%)
Previous systemic treatments – No. (%)	
0	16 (62%)
1	10 (38%)

LDH: Lactate dehydrogenase, ULN: Upper normal limit, WHO: World Health Organization

### Mutational landscape in tumour biopsies

To assess the mutation status across genes related to cancer and angiogenesis in tumour biopsies, we applied targeted sequencing using a panel of 419 genes in fresh frozen biopsies from metastatic lesions for 22 of the 26 patients (Supplementary Fig. 1). The patients had a median of 10 mutations (range 0–21, Supplementary Table 1), and number of mutations did not correlate with treatment response. Somatic mutations were detected for all but one patient. Apart from *BRAF* and *NRAS* mutations, the only other recurrent mutations were *KMT2C* p.C1114R (P46, P57 and P61), *DDX11* p.K272M and *DDX11* p.K273R (both in P26 and P38). The two *DDX11* variants were in close genomic proximity and had similar variant allele frequencies (VAFs) (P26: p.V272M; 10.8%, p.K273R; 12.8%, P38: p.V272M; 30.4%, p.K273R; 30.4%), but were found on separate reads. Based on the similarity of VAFs one can speculate that the *DDX11* variants were biallelic in the tumor clone. These variants cannot be excluded as germline, however they do appear in the minor fraction of the cells. The three patients with lowest PFS (patient P14, P51 and P64) all harboured known cancer-related somatic hotspot mutations. Patient P14 (PFS 28 days, OS 66 days) had a loss-of-function mutation in *CDKN2A* (p.D108Y)^13^, a gain-of-function mutation in the catalytic domain of *IDH1* (p.R132C)^14^ and a stabilizing mutation in *CTNNB1* (p.S45F)^15^. Patient P51 (PFS 9 days, OS 82 days) had a kinase activating mutation in *PIK3CA* (p.H1047L). Patient P64 (PFS 28 days, OS 203 days) had an inactivating mutation in *CDKN2A* (p.P114L)^16^, a loss-of-function mutation in the DNA-binding domain of *TP53* (p.S241F)^17–20^, and an activating mutation of *RAC1* (p.P29S)^21^ known to increase PD-L1 expression significantly and giving resistance to RAF inhibitors *in vitro*^22,23^. Furthermore, all patients with ≥ 2 mutations in angiogenesis-associated genes had progressive disease (median PFS: 54 days vs 68 days, OS: 188 days vs 465 days (not statistically significant, Supplementary Table 2)).

### BRAF and NRAS status in tumour biopsies

*BRAF* exon 15 and *NRAS* exon 1 and 2 were investigated by Sanger sequencing of formalin fixed paraffin embedded (FFPE) primary tumors from time of diagnosis, and metastatic tumors collected before inclusion in the clinical trial (Table 2). For *BRAF*, p.V600E was most frequent (n = 11), followed by p.V600K (n = 2) and p.V600D (n = 1). One patient was positive for *BRAF* p.V600E in the primary tumour, but positive for p.V600D in the metastasis. One patient had both *BRAF* codon 600 and *NRAS* codon 12 mutations. For *NRAS*, mutations were as follows; p.Q61R (n = 4), p.Q61K (n = 4), p.Q61L (n = 1) and p.G12C (n = 1) (Table 2).

**Table 2:** *BRAF* and *NRAS* mutations detected by Sanger, NGS and ddPCR.

Patient	Tumour - Sanger primary lesion	Tumour - Sanger metastatic lesion	Tumour - NGS^§^ metastatic lesion	cfDNA - ddPCR
P14	N.A.	N.A.	*NRAS* p.Q61K	*NRAS* p.Q61K
P19	N.A.	*BRAF* p.V600E	*BRAF* p.V600E	*BRAF* p.V600E
P26	N.A.	Wt	*BRAF* p.V600E	*BRAF* p.V600E
P31	N.A.	*BRAF* p.V600E	*BRAF* p.V600E	*BRAF* p.V600E
P32	N.A.	*NRAS* p.Q61K	*NRAS* p.Q61K	*NRAS* p.Q61K
P38	*BRAF* p.V600E	*BRAF* p.V600E	Wt	*BRAF* p.V600E
P41	*BRAF* p.V600E, *NRAS* p.G12C	N.A.	*BRAF* p.V600E Wt, *NRAS* p.G12V	*BRAF* p.V600E N.D., *NRAS* p.G12V
P42	*BRAF* p.V600K	*BRAF* p.V600K	Wt	*BRAF* p.V600K
P43	N.A.	*NRAS* p.Q61R	N.A.	*NRAS* p.Q61R
P44	*NRAS* p.Q61R	*NRAS* p.Q61R¤	*NRAS* p.Q61R¤	*NRAS* p.Q61R
P46	N.A.	*BRAF* p.V600E¤	*BRAF* p.V600E¤	*BRAF* p.V600E
P50	*BRAF* p.V600K	N.A.	N.A.	*BRAF* p.V600K
P51	N.A.	*BRAF* p.V600E	*BRAF* p.V600E	*BRAF* p.V600E
P53	Wt	N.A.	*NRAS* p.Q61K	*NRAS* p.Q61K
P57	Wt	*NRAS* p.Q61K	*NRAS* p.Q61K	*NRAS* p.Q61K
P58	*NRAS* p.Q61K	*NRAS* p.Q61K	*NRAS* p.Q61K	*NRAS* p.Q61K
P60	*NRAS* p.Q61L	*NRAS* p.Q61L	*NRAS* p.Q61L	N.D.
P61	*NRAS* p.Q61R	N.A.	*NRAS* p.Q61R	N.D.
P62	*BRAF* p.V600E	*BRAF* p.V600D	*BRAF* p.V600D	*BRAF* p.V600D
P64	*NRAS* p.Q61K	Wt¤	*NRAS* p.Q61K¤	*NRAS* p.Q61K
P65	*NRAS* p.Q61R	N.A.	*NRAS* p.Q61R	*NRAS* p.Q61R
P66	*BRAF* p.V600E	N.A.	*BRAF* p.V600E	*BRAF* p.V600E
P67	*BRAF* p.V600E^#^	N.A.	N.A.	N.D.
P68	*BRAF* p.V600E	N.A.	N.A.	*BRAF* p.V600E
P69	*BRAF* p.V600E	*BRAF* p.V600E	*BRAF* p.V600E	*BRAF* p.V600E
P70	*BRAF* p.V600E	N.A.	*BRAF* p.V600E	*BRAF* p.V600E

^§^Not identical lesion as investigated by Sanger unless otherwise stated. ^#^Mutation not detected upon reanalysis of biopsy sample by ddPCR. ¤ Matched sample with metastatic lesion analyzed by Sanger N.A: DNA not available for analysis, N.D.: no detectable *BRAF/NRAS* ctDNA

NGS was performed on fresh frozen tumour metastasis biopsies for 24 of the patients. *BRAF* or *NRAS* hotspot mutations were found in 20 of 24 patients (*BRAF* p.V600E (n = 8) and p.V600D (n = 1); *NRAS* p.Q61K (n = 6), p.Q61R (n = 3), p.Q61L (n = 1) and p.G12V (n = 1)). The two methods identified in total 15 patients positive for *BRAF* hotspot mutations and 12 patients positive for *NRAS* hotspot mutations (Table 2). Discrepancies were found for five patients when comparing Sanger and NGS (Table 2). This was assigned to four potential reasons; 1) the suspected primary tumor was collected 2–4 years before the metastatic lesion, and we cannot exclude that the patient had more than one primary tumour, 2) different tumour content between biopsies from primary and metastatic lesions, 3) different detection limits of NGS and Sanger sequencing, and 4) the fact that metastatic lesions in melanoma can be heterogeneous with respect to driver mutations^24^.

### BRAF and NRAS status in ctDNA

**Figure 2:**
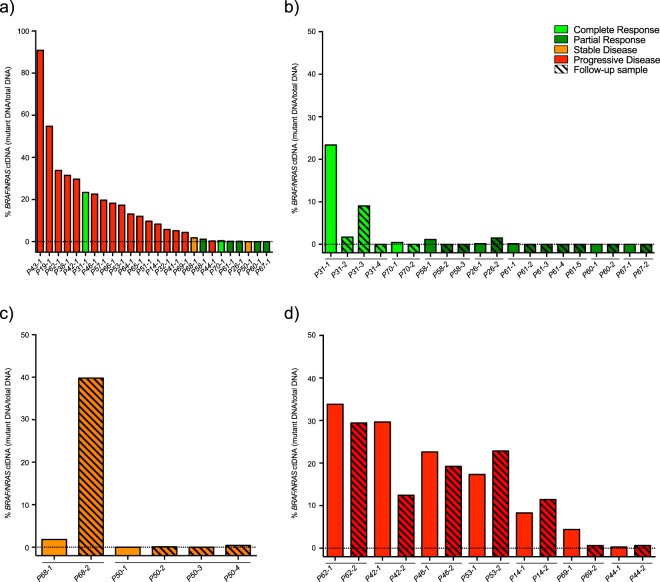
Fractional abundances of ctDNA in patients with progressive disease compared to patients responding to therapy. (**a**) All 26 patients were evaluated for ctDNA by measuring *BRAF* p.V600, *NRAS* p.Q61 and p.G12 hotspot mutations by ddPCR. ctDNA could be detected in the pre-treatment sample for all patients except P50, P60 and P67. (**b**) Follow-up samples for responding patients (CR and PR) showed the lack of ctDNA for P60 and P67, low fractional abundances detected for P26, P58 and P61, and the complete disappearance of ctDNA in P31 and P70. (**c**) Patients with stable disease had either stable low, or non-detectable ctDNA (P50), or a pronounced increase at progression (P68). **d)** Pre-treatment and follow-up samples from patients with progressive disease. (Black stapled line indicates 0% ctDNA)

To evaluate whether ctDNA was detectable in plasma, cell free DNA (cfDNA) from Sanger or NGS-identified *BRAF/NRAS* mutation-positive patients was obtained at treatment start (n = 26 patients, range 0 to 310 days before treatment, median 3 days) and at follow up (n = 16 patients) with a total of 50 samples; Fig. 1a. Using mutation specific ddPCR-assays, ctDNA was detected in 23 of 26 patients (13 *BRAF* and 10 *NRAS* (Fig. 2, Table 2, Supplementary Table 3). This gives an 88.5% concordance with the sequencing results of the biopsied tumours. Diagnostic tumour biopsy FFPE DNA was available for one of the ctDNA negative patients (P67), and ddPCR analysis did not confirm the *BRAF* p.V600E detected by Sanger (802 wildtype copies detected, 0 mutant copies detected). P60 progressed 43 months after treatment initiation and the samples investigated were obtained 10 months prior to treatment and at treatment start. Low levels of wild-type copies were detected indicating low cfDNA concentration before and at inclusion (pre-inclusion; 450 copies, treatment start; 838 copies), however samples with similar cfDNA concentration and number of wild-type copies could be found to be ctDNA positive (e.g. P14, P31, P41, P53).

### BRAF and NRAS ctDNA quantification

**Figure 3:**
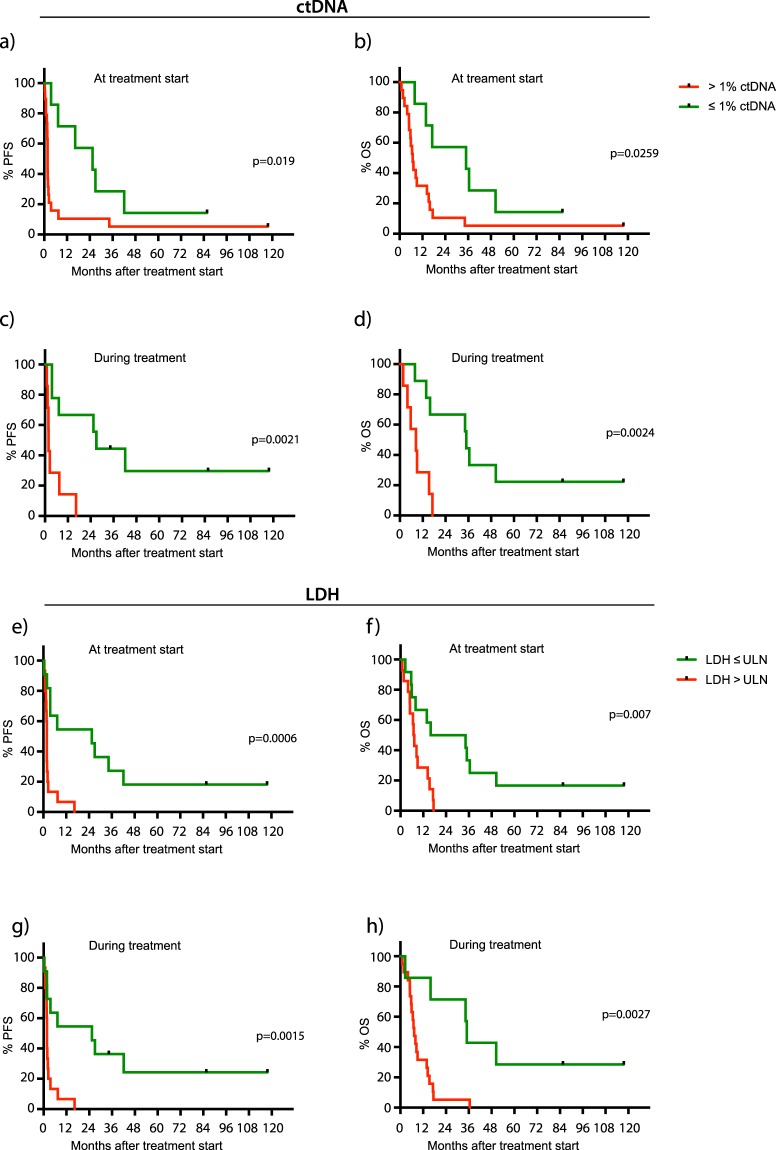
Progression free survival and overall survival in association with ctDNA (*BRAF* p.V600, *NRAS* p.G12 and p.Q61 mutations) and LDH levels. Patients with ctDNA below 1% at treatment start had significantly longer (**a**) PFS (p = 0.019) and (**b**) OS (p = 0.026) compared to patients with higher ctDNA. Patients with ctDNA levels receding below 1% during the treatment period had significantly longer (**c**) PFS (p = 0.002) and (**d**) OS (p = 0.002) compared to patients with stable or increasing fractional abundances of ctDNA. LDH levels within normal range at treatment start are associated with significantly longer **e)** PFS (p = 0.0006) and (**f**) OS (p = 0.007). Patients with LDH levels increasing above upper normal limits (ULN) during treatment have significantly shorter (**g**) PFS (p = 0.002) and (**h**) OS (p = 0.003). ULN 18–69 years: 105–205 U/L, > 69 years: 115–255 U/L.

To evaluate the response prediction potential of ctDNA, we calculated fractional abundance (percentage of mutation positive DNA fragments relative to total numbers of DNA fragments) for each sample. Patients with *BRAF/NRAS* ctDNA fractional abundance higher than 1% at treatment start had significantly lower PFS compared to patients with ctDNA fractional abundance ≤1% (median 54 vs 774 days, HR 2.52, p = 0.019) and OS (median 209 vs 1064 days, HR 2.44, p = 0.026) (Fig. 3a,b, Table 3, Supplementary Table 3). Furthermore, patients with ctDNA fractional abundance ≤1% within 9 cycles of treatment (day 126) had significantly longer PFS compared to patients with continued elevated ctDNA (median 820 vs 56 days, HR 6.76, p = 0.002) (Fig. 3c). Corresponding numbers for OS were median 1064 vs 256 days (HR 6.78, p = 0.002) (Fig. 3d). Additionally, radiological responders (CR, PR and SD) had significantly lower *BRAF/NRAS* ctDNA fractional abundance compared to non-responders (progressive disease; PD) both before treatment start (median 0.2% vs. 17.3%, PFS and OS p = 0.001) and during follow up (median 0% vs 12.5%, PFS and OS p = 0.049). Patients with *BRAF/NRAS* ctDNA fractional abundance ≤1% at treatment start also had higher chance of responding to therapy (achieving complete/partial response and stable disease) compared to patients with higher ctDNA fractional abundance (OR 16.94, p = 0.032)

**Table 3:** PFS and OS for ctDNA and LDH.

	Days median PFS (range)	Hazard Rate PFS (95% CI)	Log-rank p-value PFS	Days median OS (range)	Hazard Rate OS (95% CI)	Log-rank p-value OS
ctDNA > 1% at treatment start	54 (9–3579)	2.52 (1.02–6.22)	0.019	209 (30–3579)	2.44 (0.98–6.06)	0.026
ctDNA ≤ 1% at treatment start	774 (109–2608)			1064 (242–2608)		
ctDNA > 1% within 9 cycles of treatment	56 (28–497)	6.76 (1.70–26.92)	0.002	256 (53–520)	6.78 (1.68–27.37)	0.002
ctDNA ≤ 1% within 9 cycles of treatment	820 (109–3579)			1064 (242–3579)		
LDH > ULN at treatment start	54 (15–497)	5.36 (1.86–15.44)	0.001	215 (30–529)	4.43 (1.56–12.65)	0.007
LDH ≤ ULN at treatment start	774 (9–3579)			764 (76–3579)		
LDH > ULN during treatment	54 (15–497)	5.24 (1.28–21.47)	0.002	221 (30–529)	3.76 (0.94–15.10)	0.003
LDH ≤ ULN during treatment	774 (9–3579)			1064 (76–3579)		

### TERT promoter mutations

**Figure 4:**
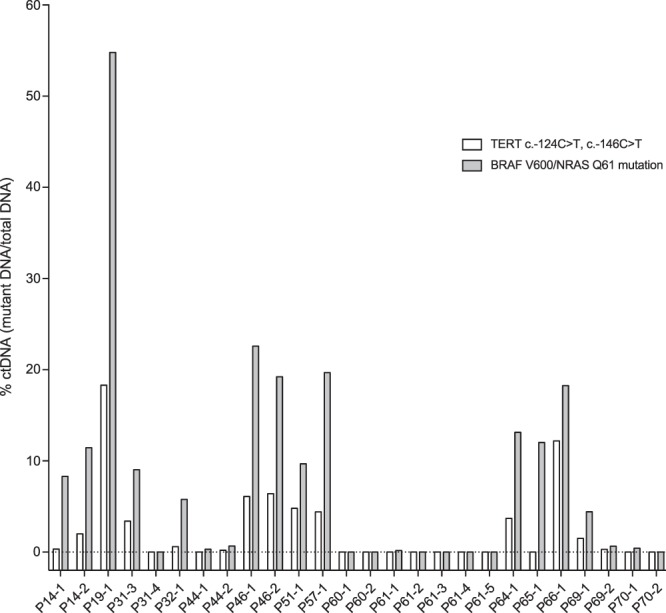
*TERT* promoter mutations measured in cell free DNA by ddPCR. *TERT* c.−124C > T or c.−146C > T (white columns) was detected in cell free DNA from 11 of 15 evaluable patients. ctDNA measured by *BRAF* p.V600, *NRAS* p.G12 and p.Q61 hotspot mutations are presented in grey for comparison. (Black stapled line indicates 0% ctDNA)

*TERT* promoter mutations are frequent in malignant melanoma^5^. In our study DNA from fresh frozen metastatic tumour biopsies were available for 19 of 26 patients. Sanger sequencing of the *TERT* promoter detected somatic mutations creating new Ets/TCF binding motifs in 15 patients (79%); 3 patients at −124 bp (C > T, chr5:1,295,228; rs1242535815) and 12 patients at −146 bp (C > T, chr5:1,295,250) (Table 4). None of the patients had both somatic variants. ddPCR detected the *TERT* promoter mutations in cfDNA for 11 of the 15 patients with biopsies positive for *TERT* c.−124C > T or c.−146C > T (14 of 26 samples tested) (Fig. 4, Supplementary Table 4). *TERT* ctDNA fractional abundances were lower than *BRAF*/*NRAS* ctDNA fractional abundances (Fig. 4). Comparison of Sanger and ddPCR in biopsy samples confirmed that this was less likely due to differences in assay efficacy, however, the genomic region is repetitive and GC rich. *TERT* ctDNA-negative samples had little or no *BRAF*/*NRAS* ctDNA (Supplementary Table 3), or had little cfDNA available. Additionally, eight patients (42%) carried the likely benign c.−245T > C germline variant (rs2853669, chr5:1,295,349 hg19 coordinate) that has been shown to reduce promoter activity in presence of other promoter mutations. In our study, *TERT* promoter variants (c.−124C > T, c.−146C > T) did not predict treatment response (OR 1.50, p = 0.657), and no correlation was found between *TERT* promoter mutations and PFS or OS.

**Table 4:** *TERT* promoter mutations.

Patient	Mutation, Sanger	Mutation, plasma, ddPCR	Polymorphism, Sanger
P14	c.−124C > T	c.−124C > T	c.−245T > C
P19	c.−146C > T	c.−146C > T	Wt
P26	Wt	—	Wt
P31	c.−146C > T	c.−146C > T	Wt
P32	c.−146C > T	c.−146C > T	c.−245T > C
P38	Wt	—	c.−245T > C
P41	Wt	—	Wt
P42	N.A.	—	N.A
P43	N.A.	—	N.A.
P44	c.−146C > T	c.−146C > T	Wt
P46	c.−124C > T	c.−124C > T	Wt
P50	N.A.	—	N.A.
P51	c.−146C > T	c.−146C > T	Wt
P53	N.A.	—	N.A.
P57	c.−146C > T	c.−146C > T	c.−245T > C
P58	Wt	—	c.−245T>C
P60	c.−146C>T	N.D.	c.−245T>C
P61	c.−146C>T	N.D.	Wt
P62	N.A.	—	N.A.
P64	c.−146C>T	c.−146C>T	Wt
P65	c.−124C>T	N.D.	Wt
P66	c.−146C>T	c.−146C>T	c.−245T>C
P67	N.A.	—	N.A.
P68	N.A.	—	N.A.
P69	c.−146C>T	c.−146C>T	c.−245T>C
P70	c.−146C>T	N.D.	Wt

N.A.: DNA not available for analysis. N.D.: no detectable *TERT* ctDNA. —: Analysis not performed.

### LDH

**Figure 5:**
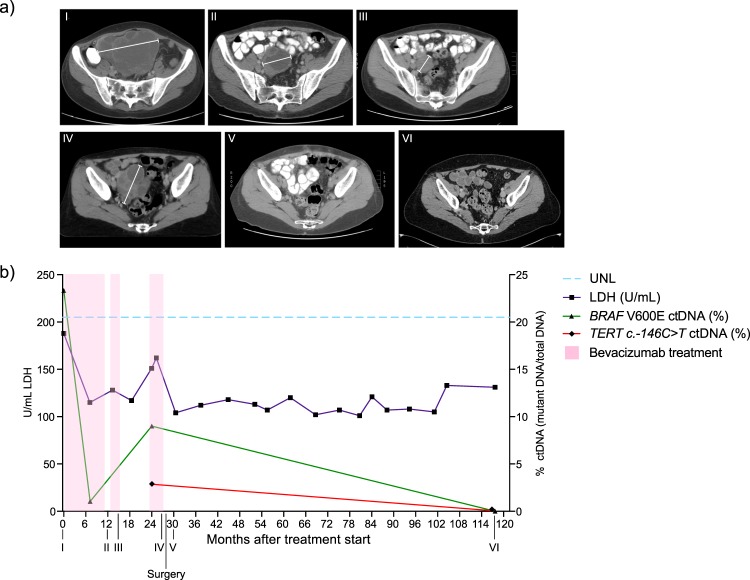
Tumour development in case P31. (**a**) CT images of the target lesion located in the ovary of P31. Scans are taken at treatment start (I), 12 months after treatment initiation (II), after 15 months (III), before resection of tumour at 27 months (IV), 2 months after surgery at 30 months (V) and after 119 months (VI). Image I and II were previously published^12^, images III-VI have not previously been published. (**b**) LDH measurements (purple) during treatment are all below upper normal limits (UNL, blue) throughout the treatment of P31. *BRAF* ctDNA (green) is high at treatment initiation, decreases after bevacizumab administration, increases at 24 months when CT images showed a large increase of the target lesion, and is not detectable at the final follow-up time point. *TERT* ctDNA (red) was available at the two last time points, and showed the same decrease as *BRAF* ctDNA. Treatment periods are indicated in pink.

To compare our *BRAF/NRAS* ctDNA findings with a more established marker for disease progression in melanoma we evaluated the levels of LDH in serum (n = 2–21 time-points per patient, Supplementary Table 5). Patients with LDH lower than upper normal limits (ULN) at therapy start showed significantly longer PFS (median 774 vs 54 days, HR 5.36, p = 0.001) and OS (median 764 vs 215 days, HR 4.43, p = 0.007) compared to patients with measurements above the normal range (Fig. 3e,f, Table 3). Samples taken during treatment showed similar results (PFS: median 774 vs 54 days, HR 5.24, p = 0.002; OS: median 1064 vs 221 days, HR 3.76, p = 0.003) (Fig. 3g,h). Furthermore, clinical responders had significantly lower LDH compared to non-responders both before treatment (PFS: p = 0.022, OS: p = 0.019), and during treatment (PFS: p = 0.008, OS: p = 0.017). Patients with normal LDH at treatment start had higher chance of responding to therapy (achieving complete/partial response and stable disease) compared to patients with LDH above ULN, but this was not statistically significant (OR 4.57, p = 0.190). ctDNA and LDH levels were positively correlated in both pre-treatment samples (r(24) = 0.805, p = 6.97 × 10^–7^) and samples taken during treatment (r(14) = 0.517, p = 0.040). Furthermore, having both ≤1% ctDNA and normal LDH at treatment start (OR 12.01, p = 0.002) was better at predicting response compared to normal levels of LDH alone, but not ≤1% ctDNA alone. However, not all patients could be discriminated by pre-treatment LDH measurements. Patient P31 (Fig. 5) is a complete responder presenting with high pre-treatment ctDNA fractional abundance (23.3%) and high tumour load according to RECIST 1.0 criteria (SLD 170 mm). After treatment with 20 courses of bevacizumab, *BRAF* ctDNA was dramatically reduced (1.7%) indicating benefit from treatment, further supported by a decrease in size of target lesions in the right ovary and both lungs measured by CT (SLD 62 mm). Whereas LDH levels fluctuate in correspondence with tumour size, it is noticeable that the levels are consistently within normal range (blue, stapled line). The following increase in ctDNA (9.03%) two years after treatment initiation was mirrored by tumour growth in the ovary (Fig. 5). The patient was operated to remove this lesion, and at follow-up 10 years after inclusion the patient is still tumour free as measured by both CT and ctDNA (0%).

### ctDNA mutation load

**Figure 6:**
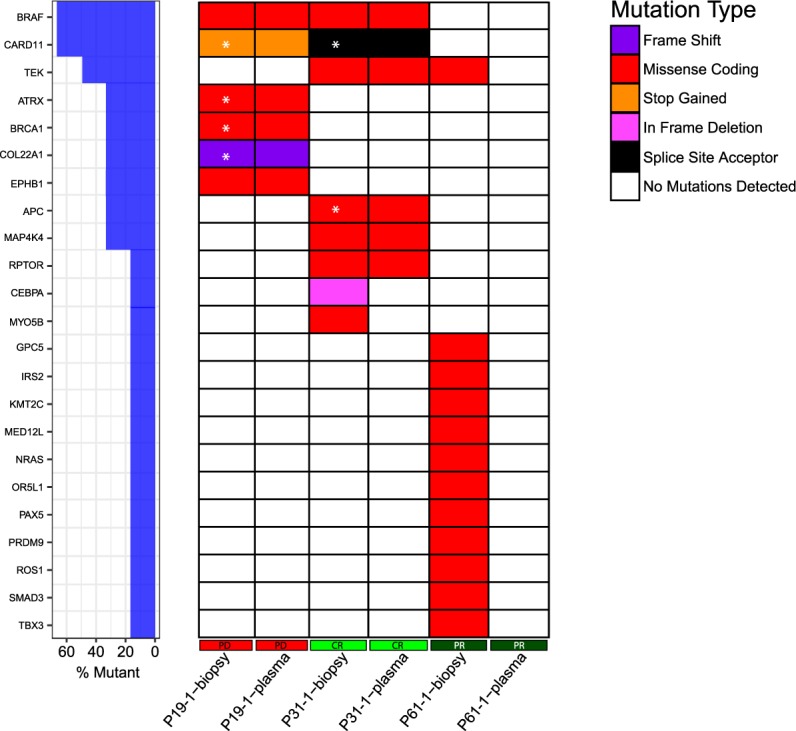
Comparison of mutations in ctDNA and metastatic lesions. Metastatic lesions were sequenced by the 419-gene panel, and ctDNA were sequenced using the 900-gene panel. Only genes included in both panels are described in the figure. For P19, all variants were discovered in both samples. For P31, two variants were not detected in plasma whereas no tumour-derived mutations were found in plasma for P61. *Variants with VAF or read depth below the defined cut-off limits in biopsy, but found at higher VAF in plasma samples.

To evaluate the potential of an extended gene panel for determining mutational load in cfDNA samples, a 900 cancer-gene panel designed by the Norwegian Cancer Genomics Consortium^25^ was used for five selected patients (Fig. 6). As genetic variants in corresponding normal DNA were not available, cancer specific mutations were defined as present in less than 1% allele frequency in population databases. Samples from two patients (P19 and P43) were evaluated to investigate patients having short PFS and high ctDNA fractional abundance, one patient (P61) was evaluated due to lack of measurable cfDNA *NRAS* mutation, and two patients (P31 and P68) were evaluated due to large decrease and increase, respectively, in CT measured tumour burden. Patients with higher mutational burden had lower PFS compared to patients with fewer mutations (Supplementary Table 6). Number of mutations detected and ctDNA *BRAF*/*NRAS* fractional abundances were correlated (r(3) = 0.988, p = 0.002). Plasma mutation frequencies were comparable by both NGS and ddPCR (Supplementary Table 7), but ddPCR showed higher sensitivity.

**Figure 7:**
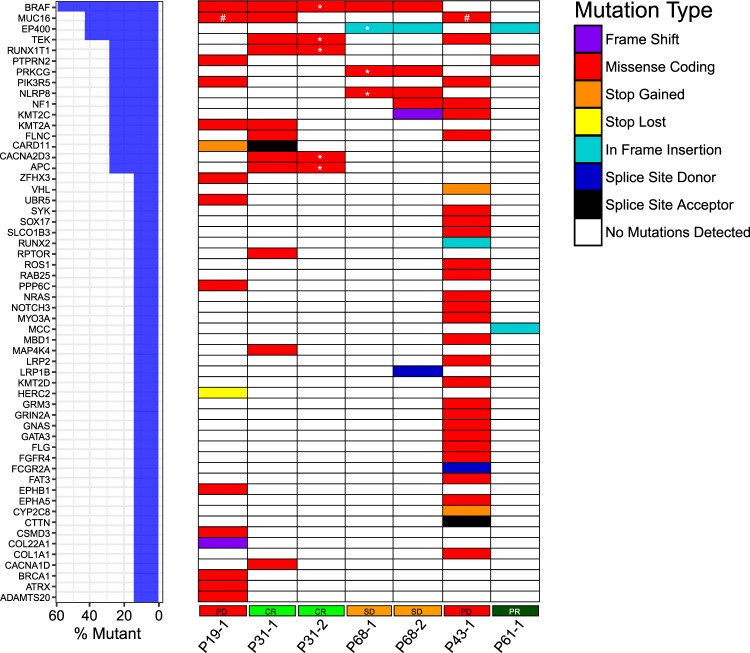
Mutations in ctDNA detected by 900-gene NGS cancer panel. The NCGC developed 900-gene NGS cancer panel was used to investigate mutational burden of plasma samples. Patients with progressive disease shows higher tumour burden than responding patients. *Variant was found by investigating reads in IGV, VAF 1–2%. #Gene harbours three mutations in different regions.

Comparison of mutations in plasma and mutations detected by the 419-gene panel in tumour biopsies was possible for three of the above patients. For P19 and P31, plasma samples showed higher frequency of *BRAF* p.V600E compared to the biopsy (56.6% vs 28.8%, and 25.6% vs 16.9%, respectively), suggesting invasion of normal cells in the biopsy samples. All mutations found in the biopsy of P19 were verified in plasma, including a missense mutation of the putative tumour suppressor *PPP6C* (p.R264C)^26^ that is sensitive to Aurora Kinase inhibition^27^ (Supplementary Table 6, Fig. 7). For patient P31, two low frequent variants were found exclusively in the tumour biopsy (*CEBPA*: p.188_189del, VAF 10.3%; *MYO5B*: p.V1703A, VAF 10.1%).

For patient P61, no variants present in the biopsy were found in plasma (Fig. 6). Of the three variants found in plasma (Fig. 7, Supplementary Table 6), all were confirmed somatic in COSMIC (*EP400, MCC, PTPRN2*), and the *EP400* variant was also seen in P68. A selection of 10 infrequent single nucleotide polymorphisms (SNPs) were compared between the biopsy and plasma sample for patient P61, confirming that the samples were from the same patient. The sample was below detection levels for *NRAS* p.Q61R ctDNA (0.05%), explaining the lack of tumour specific mutations found in plasma by the 900-gene panel. This patient did not progress until 820 days after the time-point for plasma NGS analysis, and all continuous blood sample time-points (n = 4, Fig. 1) were negative for *NRAS* p.Q61R ctDNA by ddPCR (Supplementary Table 3).

Patient P43 with the highest ctDNA fractional abundance in this study (Fig. 7, Supplementary Table 6), presented with the highest mutational load (n = 33), including missense mutations in *GRM3* (p.D257N) and *PIK3R5* (p.R741Q). Contribution of germline variants cannot be excluded, however 14 variants were verified as somatic as they are not present in any population databases. The two evaluated clinical responders, P31 (CR) and P61 (PR), both had mutations in *TEK* exon 10. Interestingly, P31 that had a significant reduction in ctDNA (from 23.4% to 1.7%) and tumour load (tumour volume reduction of 62%), also showed a reduction from 12 mutations to no mutations above VAF cut off-limits at corresponding time-points (Fig. 7, first and second time-point). An opposite pattern was observed for patient P68 that had an increase in ctDNA (from 1.8% to 39.8%) and tumour load (sum of largest diameters (SLD) from 135 to 158 mm with two new lesions) from first to second sample time-point. In plasma all four variants found in the first sample had an increase in VAF at progression of disease, and three new variants were identified at this point.

## Discussion

ctDNA has the potential to play an important part in cancer precision medicine^28^. Particularly for cancers where new biopsies are difficult to obtain, the analysis of ctDNA can provide a tool for identifying treatment targets and monitoring treatment response, minimal residual disease and relapse. With the increasing numbers of clinical trials investigating the role of bevacizumab in cancer (ClinicalTrials.gov Identifiers NCT00790010, NCT03743428, NCT02884648, NCT03836066), commonly in combination with other drugs^29–33^, the discovery of an early biomarker for treatment response, using a method that allows repetitive, minimal invasive testing at low cost, would be of high value.

CtDNA investigations can range in scale from analysis of single mutations to whole-genome analyses. Monitoring treatment response by multigene analysis using massive parallel sequencing of gene-panels or exomes has the advantage of being less dependent on identifying early mutational events. It also permits detection of subclonal tumour response and progression, and can identify treatment targets and mutational load. It is however costly, time consuming and has lower sensitivity compared to ddPCR. As approximately 70% of malignant melanoma patients have a hotspot mutation in *BRAF* or *NRAS*^34^, and the mutation being an early event in most cases, single gene analysis is suitable for monitoring these patients during the treatment course. ddPCR provides the necessary sensitivity^35^, is fast, robust and cheap, and therefore well suited for future diagnostics.

A slight discrepancy was found between mutation profiles obtained by Sanger sequencing and NGS (Table 2). As NGS is more sensitive than Sanger, *BRAF* p.V600E for P26 and *NRAS* p.Q61K for P53 were only detected by NGS. Additionally, high degree of normal cell-infiltration of some biopsies cannot be excluded as Sanger results for P38 and P42 could not be validated by NGS despite sufficient read depth (312 and 184 reads at *BRAF* codon 600, respectively). Furthermore, *BRAF* p.V600E for P41 could not be verified by NGS nor ddPCR (read depth 259).

In our study we used ddPCR to investigate the clinical validity of ctDNA measurements in melanoma patients treated with bevacizumab monotherapy by quantifying *BRAF*, *NRAS* and *TERT* promoter mutations in plasma. We showed that both normal LDH and ≤1% *BRAF*/*NRAS* positive ctDNA before and during treatment reflected response to therapy by increased PFS and OS, and that the levels of ctDNA and LDH correlated, the latter finding being supported by research by Wong *et al*.^36^ and Gray *et al*.^37^. Pre-treatment ctDNA has previously been found to not predict survival in malignant melanoma^38^, but we found that ≤1% ctDNA at inclusion was a positive predictor for response to bevacizumab and that ctDNA was a better predictor than LDH. Diem and colleagues investigated LDH as prognostic marker for metastatic melanoma patients treated with anti-PD-1 therapy, and concluded in concordance with our study that elevated levels at treatment start resulted in significantly shorter OS^39^. An association between LDH and PFS is also found in patients treated with mitogen-activated protein kinase inhibitors, suggesting this to be a universal mechanism independent by drug class^36^. Herbreteau *et al*. showed that detection of ctDNA after two weeks of treatment was 100% correlated with progressive disease in melanoma patients treated with anti-PD1 immunotherapy^40^. Felix *et al*. showed that low LDH at 3 and 6 weeks after ipilimumab treatment is significantly associated with increased OS^41^. However, normal LDH levels resulted in false prediction of treatment response in patients with progressive disease in our study, and did not reflect the change in tumour burden as accurately as ctDNA. This finding is in concordance with previous studies investigating BRAF inhibition and immune checkpoint blockade therapy^42,43^.

Recently, McEvoy and colleagues showed that detection of *TERT* mutation positive ctDNA was predictive for PFS (n = 15)^44^. A trend for increased melanoma-specific survival in *TERT* wild-type patients has been reported in larger studies^4^, and others have found that patients with mutations have shorter disease-free survival^7^. In our study ctDNA *TERT* promoter mutations were not correlated to PFS or OS. McEvoy also showed that for a substantial fraction of the patients with mutation positive tumours, *TERT* mutations could not be detected in plasma samples^44^, reflecting our findings. In addition, we observed that the fractional abundance of *TERT* was consistently lower that *BRAF/NRAS*, a finding supported by Calapre *et al*.^45^ and Wong *et al*.^36^. This could indicate that only a subclone of the tumour contains *TERT* promoter mutations, or that false low VAFs due to technical issues caused by GC rich gene regions makes detection of this variant difficult in plasma.

In ctDNA detected by the expanded 900 cancer-gene panel^25^, we observed that patients with higher mutational burden had lower PFS compared to patients with fewer mutations. This was however only based on five patients, and the observation therefore needs to be supported in a larger cohort to conclude whether our results reflects mutational burden or is a consequence of higher levels of cfDNA and ctDNA in patients with low PFS.

Whereas high mutation load is associated with treatment response in melanoma patients treated with adoptive T-cell therapy^46^, correlating with improved OS after anti-PD-1 therapy (pembrolizumab or nivolumab)^47^, no such association has been explored for anti-angiogenesis therapies. Relevant to the angiogenic effects of bevacizumab, all patients harbouring two or more mutations in angiogenesis-related genes had progressive disease (not statistically significant). Additionally, the patients with shortest PFS had several known pathogenic somatic mutations in metastatic lesions. Particularly *IDH1* p.R132C and *CTNNB1* p.S45F, as well as *TP53* p.S241F and *RAC1* p.P29S are likely pathogenic variants. *RAC1* p.P29S is an oncogenic hotspot in melanoma^48^, resulting in a spontaneous activation of this GTPase^21^ giving resistance to vemurafenib and dabrafenib^23^. Patients with this mutation are however found to have upregulated PD-L1, suggesting they might benefit from immunotherapy^22^. *IDH1* p.R132C is a hotspot mutation commonly found in acute myeloid leukaemia, but has more recently been identified in melanoma^49^, and frequently co-occurs with *NRAS* mutations^50^. The mutation alters cancer cell differentiation and results in metabolic reprogramming (reviewed in^51^). Several IDH1 inhibitors have been developed, and are undergoing phase I clinical trials reducing 2HG levels produced by the mutant protein to normal physiological levels (reviewed in^52^).

In treatment monitoring, single gene analysis require identification of an early event in the tumour and for melanoma *BRAF/NRAS* hotspot mutations are good candidates. This is supported by a high concordance between *BRAF/NRAS* mutations in plasma and biopsy (88.5%, Table 2), between NGS VAFs and ddPCR fractional abundances (Supplementary Table 7), and is in agreement with previous studies^53^. *TERT* promoter mutations are on the other hand present at lower fractional abundance (Table 4, Supplementary Table 4) and show lower concordance to tumour biopsy (73.3%), both in our and other studies^45^. Due to high GC content of the *TERT* promoter, and this being a chronologically later mutational hit, we believe *BRAF* p.V600 and *NRAS* p.Q61/p.G12 variants to be the best pharmacodynamic biomarkers to monitor treatment response in mutation positive malignant melanoma patients. Furthermore, the level of ctDNA detected by ddPCR correlates to tumour burden^54^, and studies show that undetectable ctDNA at baseline or within 8 weeks of treatment is an independent marker of response and prolonged survival in melanoma patients^38^. Collectively, this could indicate that ctDNA measurements may not only be a predictive marker, but also a prognostic marker indicating which patients would benefit from closer follow-up by CT, and potentially contribute to reducing number of CT analyses needed for patients with undetectable ctDNA.

To conclude, ddPCR of *BRAF* and *NRAS* hotspot mutations in plasma showed high concordance with NGS and Sanger sequencing of biopsy samples, and ctDNA and LDH levels reflected disease progression in the majority of patients. Moreover, low level of detected ctDNA at inclusion (≤1%) was a positive predictor for response to bevacizumab (odds ratio 16.94) and was a better predictor for response compared to LDH. NGS analyses showed that patients having mutations in two or more angiogenesis-relevant genes progressed earlier compared to patients with fewer mutations, however this was not statistically significant. No other novel genetic biomarkers for response to this drug were found. Our data support the clinical validity for ctDNA in monitoring treatment response and indicate that number of CT scans might be reduced for sub-group of patients. To confirm these findings, a randomized clinical trial with paired CT and ctDNA plasma sampling is ongoing (NCT02872259). For other clinical trials investigating the utility of bevacizumab in cancer treatment, we suggest ddPCR analyses monitoring ctDNA to be of higher clinical benefit compared to mapping the tumour mutational profile.

## Methods

### Study design

All participating patients provided signed informed consent before enrolment. Fifty-two patients were screened between April 2005 and August 2009 for this phase II, open-label, single-arm, single institution clinical trial for bevacizumab treatment (ClinicalTrials.gov Identifier: NCT00139360, 31/08/2005), performed at the Haukeland University Hospital, Bergen, Norway. Thirty-five patients met the inclusion criteria and were enrolled as described previously^12^. In short, each treatment cycle consisted of bevacizumab 10 mg/kg IV on day 1 in a 2-weekly schedule. Drug toxicity was assessed after each cycle; response rate was evaluated after every 4 cycles. Patients with disease progression or unmanageable toxicity were discontinued and offered further melanoma treatment at the clinician’s discretion. For the current study, the 26 patients with tumour biopsies positive for *BRAF* p.V600 or *NRAS* p.Q61 mutations were included (Tables 1 and 2). PFS and response rates per August 2011 were previously used as endpoints in the study by Schuster *et al*.^12^, but updated PFS, OS and response (Fig. 1) in addition to not previously published data on LDH is included in this study. Blood was collected in EDTA-tubes at treatment start, follow-up, and/or relapse for 26 patients, and immediately centrifuged at 4 °C, 1600 g. cfDNA was extracted from 2–3 mL plasma using the QIAamp DSP Circulating Nucleic Acid Kit (Qiagen) as recommended by the manufacturer. The study was conducted in accordance with the Declaration of Helsinki and the International Conference on Harmonization of Good Clinical Practice, and the protocol was approved by the Regional Ethics Committee of Western Norway and the Norwegian Medicines Agency (REK2012/910).

### Response assessment

All patients were in clinical and/or radiological progression at the time of inclusion (0 to 19 days before treatment start, median 3.5 days). The primary endpoint was objective response (OR) defined as complete response (CR) or partial response (PR) according to RECIST^55^ as well as disease control (DC) defined as CR + PR and including stable disease (SD) for more than 6 months. Briefly, response definitions were based on measurements of the longest diameter of target lesions; for CR, the target lesion was completely disappeared, for PR the sum of target lesion diameters decreased by ≥30% and for progressive disease (PD) the sum of target lesion diameters increased by ≥20%. OR and DC were calculated on the basis of investigator assessment. P43 had and P51 clinical disease progression before first radiological progression and was defined as having PD. Thus, best overall response (BOR) was not available for these patients. Image I and II of Fig. 5a have previously been published as part of a figure by Schuster *et al*.^12^, but is updated and revised in our study. Time to progression was defined as the time from enrolment to disease progression or death due to melanoma.

### Tissue sampling and sanger sequencing

Tumour tissue was acquired as core biopsies or surgical biopsies, and either snap frozen in liquid nitrogen or fixed in formalin and cast in paraffin immediately upon dissection. DNA was extracted from tumour tissue manually dissected from three paraffin sections (10 μm) using the E.Z.N.A Tissue DNA Kit (Omega Bio-Tek, Inc.,). Due to limited metastatic tissue, Sanger was not performed on the same biopsies as NGS (exceptions seen in Table 2). The DNA was screened for mutations in *BRAF* (NM_004333) exon 15, as well as *NRAS* (NM_002524) exon 1 and 2, by direct Sanger sequencing after PCR amplification (primers are described previously^56–58^). Mutations in the *TERT* promoter (NM_198253) were investigated in DNA from fresh frozen tumour metastasis biopsies by direct PCR amplification and Sanger sequencing. PCR conditions and primers are described in Supplementary Materials.

### NGS

Genomic DNA was isolated from 22 fresh frozen tumour metastasis biopsies and matched normal controls from whole blood using QIAmp DNA Mini kit (Qiagen) according to the manufacturer’s instructions. Tissue from the metastasis occurring closest to time of inclusion was used for NGS. Targeted massive parallel sequencing of 419 genes was performed for tumour and matched normal DNA for each patient. These 419 genes represented a panel of 360 cancer related genes previously described^59^, extended with 59 angiogenesis related genes (Supplementary Table 8). Gene panel specific sequencing libraries were generated by hybridisation to custom RNA baits (Agilent Technologies) according to the Agilent SureSelect protocol, and sequenced paired-end (2 × 75 bp) on an Illumina MiSeq instrument. To explore the mutational profile present in plasma derived cell free DNA (cfDNA) from 5 patients, an extended, custom SureSelect (Agilent Technologies) in-solution capture panel (NCGC 900) developed by the Norwegian Cancer Genomics Consortium was used to enrich for exons of 900 cancer-related genes, selected promoters and introns frequently involved in fusions as described previously^25^. Libraries were constructed using the ThruPLEX Plasma-Seq kit from Takara, captured using the Agilent SureSelect protocol and sequenced paired-end (2 × 100 bp) on an Illumina HiSeq. 2500 (Illumina Inc.) using TruSeq SBS v3 chemistry.

### Mutation calling

#### 419-cancer and angiogenesis-gene panel

Sequence reads were aligned to the human reference genome (NCBI build 37) using BWA^60^. Subsequent mutation calling was performed by Illumina’s MiSeq reporter software v2.2 using default settings. To exclude germline SNPs, all called tumour variants with a variant allele frequency in matched normal DNA > 0.05 were removed. Further, a set of additional post processing filters were applied in order to remove false positive calls. These included; sequencing depth at < 1000 × , total read depth > 10x and absolute number of reads carrying the variant ≥ 3.

Further, mutations were subject to manual inspection in Integrative Genomics Viewer (IGV) and likely false positives were removed from the data set based on variant position in read and mapping quality of variant-bearing reads. All called variants were annotated using Annovar and variants included in further analyses were restricted to those causing amino acid changes within the 419-gene panel.

#### NCGC 900 cancer-gene panel

Real-time analysis and base calling was performed using Ilumina’s software packages HSC2.0.2/RTA1.17.21.3 and raw reads were processed with Illumina CASAVA (v 1.8.2) to filter out low-quality reads. Variants present in the 1000 Genomes Project or gnomAD ≥ 0.01 were filtered out. Cut-off was set at alternative allele frequency > 10% and coverage 100. Sequence reads were aligned to the human reference genome (NCBI build 37) using BWA. As matched normal samples were not available, the following criteria were used to remove germline variants; > 1% frequency in the 1000 Genomes Project or gnomAD, variants found as germline in the 419-gene panel, variants found with VAF 0.4–0.6 or 0.8–1.0 in two samples from the same patient. All potential rare germline mutations are therefore not filtered out.

### Droplet digital PCR

ctDNA was assessed using ddPCR™ Mutation Detection Assay: *NRAS* p.Q61R, *NRAS* p.Q61L, *NRAS* p.G12V, *BRAF* p.V600K, *BRAF* p.V600D; PrimePCR™ Mutation Assay: *NRAS* p.Q61K, *NRAS* WT for p.Q61K, *BRAF* p.V600E, *BRAF* WT for p.V600E; ddPCR™ Expert Design Assay: *TERT* C288T_88, *TERT* C250T_88 (all from Bio-Rad). All samples were run in duplicate. Samples generating a total of ≤ 2 droplets positive for the mutation assay were defined as having *ctDNA not detected*. Droplets were generated using the QX200 Droplet Generator (Bio-Rad), and the PCR reaction is described in Supplementary Material. Droplets were read using the QX200 Droplet Reader (Bio-Rad) and data was analysed using QuantaSoft version 1.7.4. Results are presented as % ctDNA (number of droplets positive for mutant-assay/total number of mutant- and wt-assay positive droplets) defined as fractional abundance.

### Statistical methods

PFS and OS related to ctDNA and LDH were assessed using the Log-rank (Mantel-Cox) test (GraphPad Prism Version 6.0d). Hazard ratios were calculated by Cox Multivariate regression and odds ratios by Logistic Regression (IBM SPSS Statistics Version 24.0.0.1); Contingency between responding groups was analysed by the Pearson’s Chi-squared test describing two-tailed p-values (GraphPad Prism) and correlation between ctDNA and LDH was performed by Pearson’s bivariate analysis describing two-tailed p-values (IBM SPSS Statistics).

## Supplementary information

Supplementary Information

Supplementary Table 1

Supplementary Table 2

Supplementary Table 3

Supplementary Table 4

Supplementary Table 5

Supplementary Table 6

Supplementary Table 7

Supplementary Table 8

## References

[1] Calapre L, Warburton L, Millward M, Ziman M, Gray ES (2017). Circulating tumour DNA (ctDNA) as a liquid biopsy for melanoma. Cancer Lett.

[2] Lee JH, Choi JW, Kim YS (2011). Frequencies of BRAF and NRAS mutations are different in histological types and sites of origin of cutaneous melanoma: a meta-analysis. Br J Dermatol.

[3] Huang FW (2013). Highly recurrent TERT promoter mutations in human melanoma. Science.

[4] Hugdahl E, Kalvenes MB, Mannelqvist M, Ladstein RG, Akslen LA (2017). Prognostic impact and concordance of TERT promoter mutation and protein expression in matched primary and metastatic cutaneous melanoma. Br J Cancer.

[5] Bai X (2017). MAPK Pathway and TERT Promoter Gene Mutation Pattern and Its Prognostic Value in Melanoma Patients: A Retrospective Study of 2,793 Cases. Clin Cancer Res.

[6] Seynnaeve B (2017). Genetic and Epigenetic Alterations of TERT Are Associated with Inferior Outcome in Adolescent and Young Adult Patients with Melanoma. Sci Rep.

[7] Nagore E (2016). TERT promoter mutations in melanoma survival. Int J Cancer.

[8] Ferrara N, Hillan KJ, Gerber HP, Novotny W (2004). Discovery and development of bevacizumab, an anti-VEGF antibody for treating cancer. Nat Rev Drug Discov.

[9] Corrie PG (2018). Adjuvant bevacizumab for melanoma patients at high risk of recurrence: survival analysis of the AVAST-M trial. Annals of oncology: official journal of the European Society for Medical Oncology.

[10] Schuster C, Akslen LA, Stokowy T, Straume O (2019). Predictive value of angiogenic proteins in patients with metastatic melanoma treated with bevacizumab monotherapy. J Pathol Clin Res.

[11] Shitara K (2016). Randomized study of FOLFIRI plus either panitumumab or bevacizumab for wild-type KRAS colorectal cancer-WJOG 6210G. Cancer Sci.

[12] Schuster C (2012). Clinical efficacy and safety of bevacizumab monotherapy in patients with metastatic melanoma: predictive importance of induced early hypertension. PLoS One.

[13] Greenblatt MS (2003). Detailed computational study of p53 and p16: using evolutionary sequence analysis and disease-associated mutations to predict the functional consequences of allelic variants. Oncogene.

[14] Jin G (2012). Mutant IDH1 is required for IDH1 mutated tumor cell growth. Oncotarget.

[15] Colombo C (2013). CTNNB1 45F mutation is a molecular prognosticator of increased postoperative primary desmoid tumor recurrence: an independent, multicenter validation study. Cancer.

[16] Arap W, Knudsen ES, Wang JY, Cavenee WK, Huang HJ (1997). Point mutations can inactivate *in vitro* and *in vivo* activities of p16(INK4a)/CDKN2A in human glioma. Oncogene.

[17] Freed-Pastor WA, Prives C (2012). Mutant p53: one name, many proteins. Genes Dev.

[18] Wang W, Kim SH, El-Deiry WS (2006). Small-molecule modulators of p53 family signaling and antitumor effects in p53-deficient human colon tumor xenografts. Proceedings of the National Academy of Sciences of the United States of America.

[19] Sur S (2009). A panel of isogenic human cancer cells suggests a therapeutic approach for cancers with inactivated p53. Proceedings of the National Academy of Sciences of the United States of America.

[20] Nazeer FI (2011). p53 inhibits mRNA 3′ processing through its interaction with the CstF/BARD1 complex. Oncogene.

[21] Davis MJ (2013). RAC1P29S is a spontaneously activating cancer-associated GTPase. Proceedings of the National Academy of Sciences of the United States of America.

[22] Vu HL, Rosenbaum S, Purwin TJ, Davies MA, Aplin AE (2015). RAC1 P29S regulates PD-L1 expression in melanoma. Pigment Cell Melanoma Res.

[23] Watson IR (2014). The RAC1 P29S hotspot mutation in melanoma confers resistance to pharmacological inhibition of RAF. Cancer Res.

[24] Yancovitz M (2012). Intra- and inter-tumor heterogeneity of BRAF(V600E))mutations in primary and metastatic melanoma. PLoS One.

[25] Namlos HM (2017). Use of liquid biopsies to monitor disease progression in a sarcoma patient: a case report. BMC cancer.

[26] Hodis E (2012). A landscape of driver mutations in melanoma. Cell.

[27] Gold HL (2014). PP6C hotspot mutations in melanoma display sensitivity to Aurora kinase inhibition. Molecular cancer research: MCR.

[28] Wan JCM (2017). Liquid biopsies come of age: towards implementation of circulating tumour DNA. Nat Rev Cancer.

[29] Lindholm Evita Maria, Ragle Aure Miriam, Haugen Mads Haugland, Kleivi Sahlberg Kristine, Kristensen Vessela N., Nebdal Daniel, Børresen‐Dale Anne‐Lise, Lingjærde Ole Christian, Engebraaten Olav (2019). miRNA expression changes during the course of neoadjuvant bevacizumab and chemotherapy treatment in breast cancer. Molecular Oncology.

[30] Rini BI (2019). Atezolizumab plus bevacizumab versus sunitinib in patients with previously untreated metastatic renal cell carcinoma (IMmotion151): a multicentre, open-label, phase 3, randomised controlled trial. Lancet.

[31] Quillien V (2019). Absolute numbers of regulatory T cells and neutrophils in corticosteroid-free patients are predictive for response to bevacizumab in recurrent glioblastoma patients. Cancer Immunol Immunother.

[32] Pietrantonio F (2019). Perioperative Bevacizumab-based Triplet Chemotherapy in Patients With Potentially Resectable Colorectal Cancer Liver Metastases. Clin Colorectal Cancer.

[33] Tew WP (2018). Randomized phase II trial of bevacizumab plus everolimus versus bevacizumab alone for recurrent or persistent ovarian, fallopian tube or peritoneal carcinoma: An NRG oncology/gynecologic oncology group study. Gynecol Oncol.

[34] Sini MC (2018). Genetic alterations in main candidate genes during melanoma progression. Oncotarget.

[35] Busser B (2017). Plasma Circulating Tumor DNA Levels for the Monitoring of Melanoma Patients: Landscape of Available Technologies and Clinical Applications. Biomed Res Int.

[36] Wong, S. Q. *et al*. Circulating Tumor DNA Analysis and Functional Imaging Provide Complementary Approaches for Comprehensive Disease Monitoring in Metastatic Melanoma. 1-14, 10.1200/po.16.00009 (2017). 10.1200/PO.16.00009 35172485

[37] Gray ES (2015). Circulating tumor DNA to monitor treatment response and detect acquired resistance in patients with metastatic melanoma. Oncotarget.

[38] Lee JH (2017). Circulating tumour DNA predicts response to anti-PD1 antibodies in metastatic melanoma. Annals of oncology: official journal of the European Society for Medical Oncology.

[39] Diem S (2016). Serum lactate dehydrogenase as an early marker for outcome in patients treated with anti-PD-1 therapy in metastatic melanoma. Br J Cancer.

[40] Herbreteau G (2018). Quantitative monitoring of circulating tumor DNA predicts response of cutaneous metastatic melanoma to anti-PD1 immunotherapy. Oncotarget.

[41] Felix J (2016). Relevance of serum biomarkers associated with melanoma during follow-up of anti-CTLA-4 immunotherapy. Int Immunopharmacol.

[42] Chang GA (2016). Sensitivity of plasma BRAFmutant and NRASmutant cell-free DNA assays to detect metastatic melanoma in patients with low RECIST scores and non-RECIST disease progression. Mol Oncol.

[43] Tsao SC (2015). Monitoring response to therapy in melanoma by quantifying circulating tumour DNA with droplet digital PCR for BRAF and NRAS mutations. Sci Rep.

[44] McEvoy AC (2017). Sensitive droplet digital PCR method for detection of TERT promoter mutations in cell free DNA from patients with metastatic melanoma. Oncotarget.

[45] Calapre L (2019). Locus-specific concordance of genomic alterations between tissue and plasma circulating tumor DNA in metastatic melanoma. Mol Oncol.

[46] Lauss M (2017). Mutational and putative neoantigen load predict clinical benefit of adoptive T cell therapy in melanoma. Nat Commun.

[47] Hugo W (2016). Genomic and Transcriptomic Features of Response to Anti-PD-1 Therapy in Metastatic Melanoma. Cell.

[48] Krauthammer M (2012). Exome sequencing identifies recurrent somatic RAC1 mutations in melanoma. Nat Genet.

[49] Lopez GY (2010). IDH1(R132) mutation identified in one human melanoma metastasis, but not correlated with metastases to the brain. Biochem Biophys Res Commun.

[50] Linos K, Tafe LJ (2018). Isocitrate dehydrogenase 1 mutations in melanoma frequently co-occur with NRAS mutations. Histopathology.

[51] Mondesir J, Willekens C, Touat M, de Botton S (2016). IDH1 and IDH2 mutations as novel therapeutic targets: current perspectives. J Blood Med.

[52] Ragon BK, DiNardo CD (2017). Targeting IDH1 and IDH2 Mutations in Acute Myeloid Leukemia. Curr Hematol Malig Rep.

[53] Rowe SP (2018). From validity to clinical utility: the influence of circulating tumor DNA on melanoma patient management in a real-world setting. Mol Oncol.

[54] McEvoy AC (2018). Correlation between circulating tumour DNA and metabolic tumour burden in metastatic melanoma patients. BMC cancer.

[55] Therasse P (2000). New guidelines to evaluate the response to treatment in solid tumors. European Organization for Research and Treatment of Cancer, National Cancer Institute of the United States, National Cancer Institute of Canada. J Natl Cancer Inst.

[56] Akslen LA (2008). Mutation analysis of the EGFR-NRAS-BRAF pathway in melanomas from black Africans and other subgroups of cutaneous melanoma. Melanoma Res.

[57] Davies H (2002). Mutations of the BRAF gene in human cancer. Nature.

[58] Omholt K, Platz A, Kanter L, Ringborg U, Hansson J (2003). NRAS and BRAF mutations arise early during melanoma pathogenesis and are preserved throughout tumor progression. Clin Cancer Res.

[59] Yates LR (2015). Subclonal diversification of primary breast cancer revealed by multiregion sequencing. Nature medicine.

[60] Li H, Durbin R (2010). Fast and accurate long-read alignment with Burrows-Wheeler transform. Bioinformatics (Oxford, England).

