# Supplementary material for: ctDNA detected by ddPCR reveals changes in tumour load in metastatic malignant melanoma treated with bevacizumab
Source: Sci Rep. 2019 Nov 25;9:17471. doi: 10.1038/s41598-019-53917-5 (PMC6877652; doi:10.1038/s41598-019-53917-5)

ctDNA detected by ddPCR reveals changes in tumour load in metastatic malignant melanoma treated with bevacizumab

Rakel Brendsdal Forthun[1], Randi Hovland[2], Cornelia Schuster[3,4], Hanne Puntervoll[3], Hans Petter Brodal[1], Heidi Marie Namløs[5], Lars Birger Aasheim[6], Leonardo A. Meza-Zepeda[5,7], Bjørn Tore Gjertsen[1,3], Stian Knappskog[4,8], Oddbjørn Straume*[3,4].

[1] Department of Medicine, Haukeland University Hospital, Bergen, Norway; [2] Department of Medical Genetics, Haukeland University Hospital, Bergen, Norway; [3] Centre of Cancer Biomarkers, CCBIO, Department of Clinical Medicine, University of Bergen, Bergen, Norway; [4] Department of Oncology, Haukeland University Hospital, Bergen, Norway; [5] Department of Tumour Biology, Institute for Cancer Research, Oslo University Hospital, Oslo, Norway; [6] Norwegian Cancer Genomics Consortium, Institute for Cancer Research, The Norwegian Radium Hospital/Oslo University Hospital, Oslo, Norway; [7] Genomics Core Facility, Department of Core Facilities, Oslo University Hospital, Oslo, Norway, [8] Section of Oncology, Department of Clinical Science, University of Bergen, Bergen, Norway.

*Corresponding author

Supplementary material

Sanger sequencing

TERT PCR was done in a 20 µl reaction containing 1 µl 20 ng/µl DNA, 4 µl GC-enhancer (Applied Biosystems, Foster City, CA, USA), 10 µl AB-Amplitaq Gold 360 (Applied Biosystems) and 1 µl 10 µM primers (*TERT*_F 5’-ACGAACGTGGCCAGCGGCAG-3’ and *TERT*_R 5’-CTGGCGTCCCTGCACCCTGG-3’) to amplify a 474 bp region. PCR program was as described previously [1]. The Sanger sequence reactions were performed using the Terminator Cycle Sequencing kit, BigDye version 1.1 (Applied Biosystems). PCR program for *TERT* was as following: 95°C for 5 min, followed by 28 cycles of 95°C for 30 s, annealing at 62°C for 1 min and polymerization at 72°C for 1 min, ended by 72°C for 7 min. Reactions were analysed on an ABI PRISM® 3100 Genetic Analyzer, applying Sequencing Analysis software, version 3.7 (both from Applied Biosystems) for *BRAF* and *NRAS*, and ABI PRISM® 3730 for *TERT* promoter sequencing.

Droplet digital PCR

9 mL blood was collected in EDTA-tubes at treatment start, at follow-up, and/or at relapse for 26 patients. The tubes were centrifuged at 2500 rpm within 2 hours of collection and the supernatant was stored at -80°C. cfDNA was extracted from 2-3 mL plasma using the QIAamp DSP Circulating Nucleic Acid Kit (Qiagen) as recommended by the manufacturer and quantified using a QuBit Fluorometer (Thermo Fisher Scientific, Waltham, Massachusetts, US).

The reaction mixture (20 µl) contained ddPCR Supermix for probes (no UTP), mutant and wild type probes (ddPCR™ Mutation Detection Assay: *NRAS* p.Q61R c.182A>G (FAM/HEX), *NRAS* p.Q61L c.182A>T (FAM/HEX), *NRAS* p.G12V (FAM/HEX), *BRAF* p.V600K (FAM/HEX), *BRAF* p.V600D c.1799_1800TG>AC (FAM/HEX), PrimePCR™ Mutation Assay: *NRAS* p.Q61K c.181C>A (FAM), *NRAS* WT for p.Q61K (HEX), *BRAF* p.V600E c.1799T>A (FAM), *BRAF* WT for p.V600E (HEX), ddPCR™ Expert Design Assay: *TERT* C288T_88 (FAM/HEX), *TERT* C250T_88 (FAM/HEX); all from Bio-Rad), template (range 0.6-47 ng) and ddH_2O. All assays had a limit of detection of 0.1%, apart from the two *TERT* assays which had a limit of detection of 0.17%. For *TERT*, 0.5 M Betaine (supplier) and 1 mM EDTA (supplier) was also included in the reaction mixture. All samples

were run in duplicate. Samples generating a total of ≤2 droplets positive for the mutation assay were defined as having *ctDNA not detected*. Droplets were generated using the QX200 Droplet Generator (Bio-Rad), before transfer to PCR plates as recommended by the manufacturer. The PCR reaction was performed using a T100™ Thermal Cycler (Bio-Rad), with the following program (*BRAF* and *NRAS*): 95°C for 10 min, followed by 40 cycles of 94°C for 30 sec and 55°C for 1 min, 98°C for 10 min and 4°C ∞. Ramp rate was set at 2.5°C /sec. For *TERT* the following program was used: 95°C for 10 min, followed by 50 cycles of 96°C for 30 sec and 62°C for 1 min, 98°C for 10 min and 4°C ∞. Ramp rate was set at 2.5°C /sec. Droplets were read using the QX200 Droplet Reader (Bio-Rad) and data was analysed using QuantaSoft version 1.7.4. All runs included no-template controls, amplification controls of wild-type *BRAF*, *NRAS* and *TERT*, as well as *BRAF* (p.V600E, p.V600K and p.V600D), *NRAS* (p.Q61K, p.Q61L, p.Q61R and p.G12V) and *TERT* (c.-124C>T and c.-146C>T) positive controls. Results are presented as % ctDNA (number of droplets positive for mutant-assay/total number of mutant- and wt-assay positive droplets).

LDH

Per institutional values, upper normal limits (ULN) is 205 U/L for patients <69 years, and 255 U/L for patients older than 70 years [2].

Figure legends

Supplementary Figure 1. Mutations detected in metastatic lesions by 419-gene NGS cancer panel.

All mutations meeting the variant filter described in the material and methods section are described and colour coded according to the figure legend. # More than one variant found.
* Variants with VAF or read depth below the defined cut-off limits in biopsy, but found at higher VAF in plasma samples.

References

1 Hayward, N. K. *et al.* Whole-genome landscapes of major melanoma subtypes. *Nature* **545**, 175-180, doi:10.1038/nature22071 (2017).

2 Rustad, P. *et al.* The Nordic Reference Interval Project 2000: recommended reference intervals for 25 common biochemical properties. *Scand J Clin Lab Invest* **64**, 271-284, doi:10.1080/00365510410006324 (2004).

Supplementary Figure 1

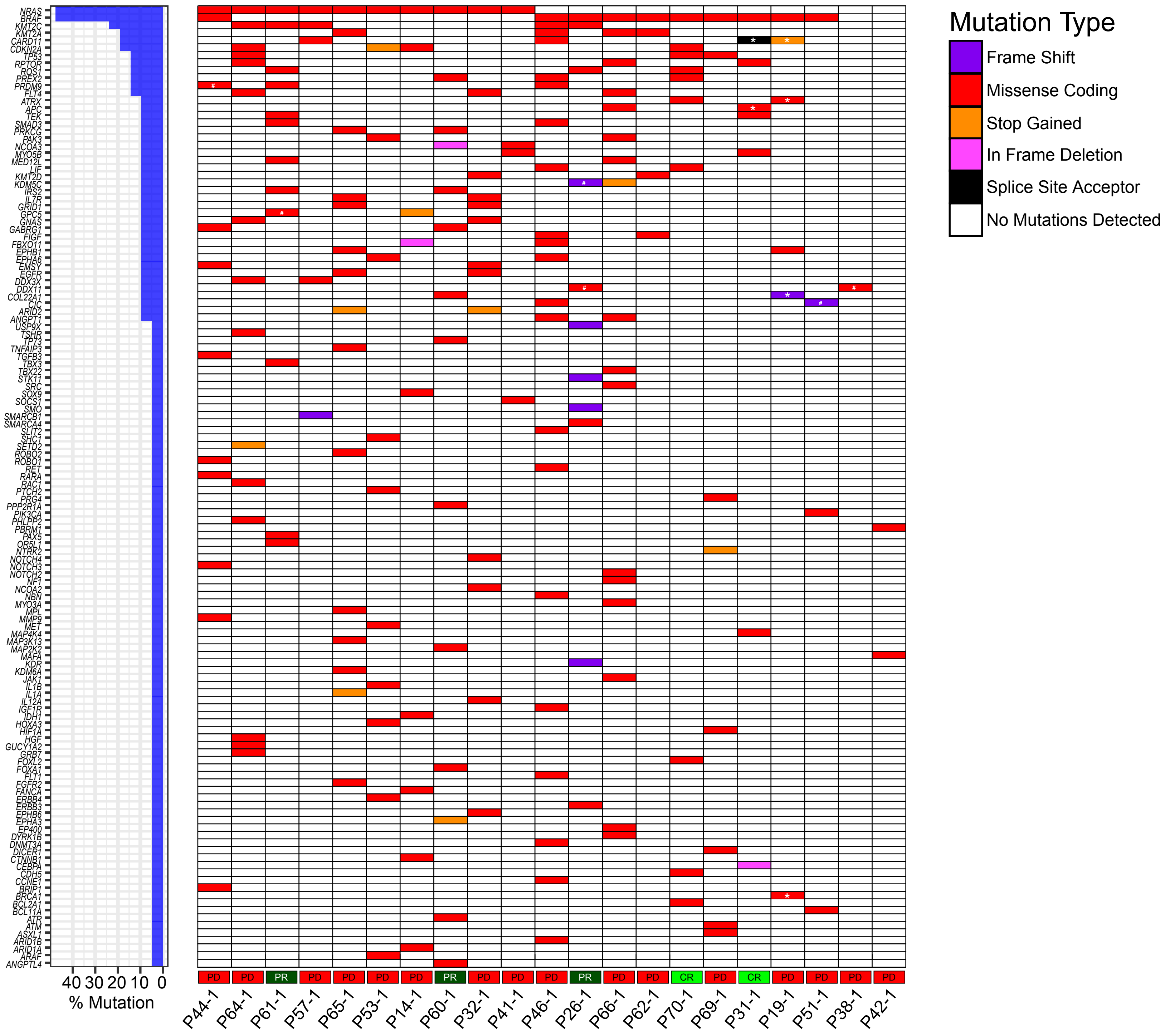

Supplement: Supplementary file 1 — Supplementary Information [file 41598_2019_53917_MOESM1_ESM.pdf]
